# From Reduction To Remediation: Sustainable Use of Algal Fibrous Mats for Silver Nanoparticle Synthesis and Dye Removal

**DOI:** 10.1002/marc.202401033

**Published:** 2025-02-20

**Authors:** Fatma Rabia Karaduman, Betül Öztürk Köksal, Ayşegül Ülkü Metin, Nesrin Horzum

**Affiliations:** ^1^ Department of Chemistry Kırıkkale University Kırıkkale 71450 Türkiye; ^2^ Nanotechnology Graduate Program İzmir Katip Çelebi University İzmir 35620 Türkiye; ^3^ Department of Engineering Sciences İzmir Katip Çelebi University İzmir 35620 Türkiye

**Keywords:** biogenic synthesis, electrospinning, nanofibers, nanoparticles, reusable photocatalyst

## Abstract

Biogenic synthesis of metal nanoparticles offers a sustainable alternative to traditional methods that often rely on toxic reducing agents, offering an environmentally friendly approach to nanoparticle production. The use of nanofibrous substrates, algal nanofibers (Polyacrylonitrile (PAN)/*Cystoseira barbata* (*Cb*)), for the reduction process enhances the efficiency of nanoparticle formation, providing a larger surface area for reaction and ensuring uniform distribution of the synthesized nanoparticles. Following the biogenic synthesis of Ag nanoparticles and their stabilization with xanthan gum (XG), the resulting PAN/*Cb*/Ag@XG nanofibrous catalyst demonstrates excellent reusability, maintaining its activity and structural integrity even after multiple cycles of use. The stabilization with XG also ensures long‐term shelf life by preventing nanoparticle aggregation. Additionally, the nanofibrous material exhibits antimicrobial activity against *E. coli* and *S. aureus*. Its dual functionality—targeting harmful pathogens while avoiding secondary pollution—positions them as a sustainable and eco‐friendly solution for advanced water purification and disinfection systems.

## Graphical abstract

Biogenically synthesized Ag nanoparticles exhibit antibacterial activity. Post‐reduction, the Ag‐decorated algal nanofibers, potentially a secondary pollutant, are repurposed for photocatalytic dye degradation, highlighting a sustainable approach for both nanoparticle synthesis and environmental remediation.

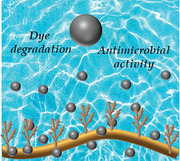

## 1 Introduction

Increasing water pollution, mainly from industrial waste, has become a major global concern. Key contributors to this pollution include industries like textiles, plastics, food processing, tanneries, cosmetics, and pharmaceuticals, which frequently release toxic chemical dyes. Many industries discharge wastewater contaminated with these dyes directly into rivers, lakes, and sewers, while in some regions, the wastewater is even used for irrigation. This practice leads to soil and crop contamination, further damaging the ecosystem and public health. The dye‐contaminated water also prevents sunlight from penetrating, which harms aquatic life by disrupting photosynthesis and oxygen production.^[^ ^1^ ^]^ The degradation of dye molecules is challenging due to their complex chemical structure, which enhances their stability.^[^ ^2^, ^3^ ^]^ Therefore, there is a growing demand for green, cost‐effective, and efficient alternatives that can not only detoxify polluted water but also reduce the overall environmental footprint of industrial processes.

Metallic nanoparticles have emerged as a promising solution because of their physical and chemical properties.^[^ ^4^ ^]^ Particularly, silver (Ag) nanoparticles stand out for their chemical stability, exceptional photocatalytic and antimicrobial capabilities, making them highly effective in water disinfection.^[^ ^5^, ^6^ ^]^ Their ability to inactivate various pathogens, including bacteria and viruses, has become increasingly important, especially during the COVID‐19 pandemic.^[^ ^7^ ^]^ By integrating dye degradation with antimicrobial properties, Ag nanoparticles provide a sustainable and efficient approach to water purification, benefiting both environmental remediation and public health. Additionally, silver nanoparticles have demonstrated remarkable potential in biomedical applications, including wound healing, drug delivery, and biosensing, due to their biocompatibility and antimicrobial properties. Their ability to generate reactive oxygen species (ROS) under light exposure enhances their potential in cancer therapy and photothermal treatments, further expanding their role in nanomedicine.^[^ ^8^ ^]^

Ag nanoparticles can be synthesized using physical, chemical, and biological approaches. Physical methods, such as laser ablation and ball milling, provide controlled particle size and purity but often require specialized equipment and high‐energy inputs. Chemical methods, including chemical reduction and sol‐gel processes, are widely used because of their simplicity and scalability. These methods involve reducing precursor salts using reducing agents like sodium borohydride or citrate, but they may leave toxic residues, requiring post‐synthesis purification. Biological methods, or green synthesis, utilize plant extracts, microorganisms, or enzymes as reducing and stabilizing agents, offering an eco‐friendly alternative with reduced environmental impact.^[^ ^9^ ^]^ Biopolymers, agricultural wastes, and animal‐derived materials also contribute to green synthesis. Among them, liquid extracts are the most commonly preferred, typically derived from different parts of plants such as leaves, flowers, fruits, peels, seeds, stems, and roots.^[^ ^10^ ^]^ Phytochemicals found in biological extracts, such as phenolic compounds (e.g., flavonoids, tannins), alkaloids, terpenoids, polysaccharides (e.g., glucose, fructose), proteins (e.g., cysteine), and vitamins (e.g., ascorbic acid), play crucial roles in the reduction and stabilization of nanoparticles.^[^ ^11^ ^]^ Algae are another alternative for Ag nanoparticle synthesis, as they not only contain the aforementioned phytochemicals but also have a rich content of pigments, including chlorophylls, carotenoids (e.g., fucoxanthin, astaxanthin), and phycobiliproteins (e.g., phycocyanin, phycoerythrin). Cyanobacterial Ag nanoparticles from *Trichodesmium erythraeum*,^[^ ^12^ ^]^ *Microchaete* sp.,^[^ ^13^ ^]^ *Oscillatoria* sp.,^[^ ^14^ ^]^ *Nostoc carneum*,^[^ ^15^ ^]^ *Spirulina platensis*,^[^ ^16^ ^]^ have been previously reported.^[^ ^17^ ^]^ Furthermore, green algae species —such as *Enteromorpha flexuosa*,^[^ ^18^ ^]^ *Ulva flexuosa*,^[^ ^19^ ^]^ *Caulerpa serrulata*,^[^ ^20^ ^]^ *Acutodesmus dimorphus*,^[^ ^21^ ^]^ *Chlorella ellipsoidea*,^[^ ^22^ ^]^ *Chlamydomonas reinhardtii*,^[^ ^23^ ^]^ *Desmodesmus* sp.,^[^ ^24^ ^]^ *Pseudopediastrum boryanum*,^[^ ^25^ ^]^ *Botryococcus braunii* ^[^ ^26^ ^]^ —are among the most commonly used. Red algae including Pterocladiella *capillacea*,^[^ ^27^ ^]^ *Hypnea musciformis*,^[^ ^28^ ^]^ *Laurencia papillosa*,^[^ ^29^ ^]^ *Turbinaria ornata*,^[^ ^30^ ^]^ *Gelidium amansii*,^[^ ^31^ ^]^ *Portieria hornemannii* ^[^ ^32^ ^]^ and brown algae such as *Sargassum vulgare* ^[^ ^33^ ^]^ and *Padina pavonia* ^[^ ^34^ ^]^ have also been explored for Ag nanoparticle synthesis.

Recent studies have highlighted the efficient degradation of dyes such as methylene blue, methyl orange, Congo red, rhodamine B, malachite green, and 4‐nitrophenol using green‐synthesized Ag nanoparticles, demonstrating their potential for effective environmental remediation.^[^ ^35^, ^36^ ^]^ Particulary, powdered or extracts of algae have been used as biofactories for Ag nanoparticles for photocatalytic remediation of dyes.^[^ ^37^ ^]^ Ag nanoparticles derived from *Sargassum serratifolium* extract^[^ ^38^ ^]^ have been utilized for the photocatalytic degradation of methyl orange, methylene blue, and rhodamine B, with *Ulva lactuca* ^[^ ^39^ ^]^ specifically targeting methyl orange. In addition to algae extracts, powdered algae are rarely used,^[^ ^22^ ^]^ as both face challenges such as low solubility, difficulty in dispersing, stability concerns, and the need for refrigeration to prevent degradation.

To mitigate these inherent challenges and promote sustainable production, the incorporation of structural frameworks or carrier substances—such as composite films, hydrogels, cryogels, and nanofibers—emerges as a promising approach. This strategy not only enhances stability and long‐term functionality but also enables the controlled formation of nanoparticles, eliminating the need for secondary centrifugation, particularly in powdered samples. To increase the surface area and gain mechanical integrity, electrospinning has become a prominent technique, enabling the production of ultrafine nanofibers. By controlling the electrospinning parameters, such as voltage, tip‐to‐collector distance, and flow rate, nanofibers with tailored properties can be fabricated for specific applications. Additionally, electrospun nanofibers offer significant advantages in terms of structural stability and flexibility, making them suitable for a wide range of industrial, environmental, and biomedical applications.^[^ ^40^ ^]^

Expanding upon the benefits of electrospinning, recent research has shifted toward “green electrospinning” as an environmentally sustainable alternative, focusing on using eco‐friendly solvents and materials, aligning with the growing demand for sustainable production methods.^[^ ^41^ ^]^ Algal nanofibers, derived from algae, have attracted considerable attention due to their biodegradability, abundance, and minimal environmental impact. While algal nanofibers are predominantly studied for biomedical applications, such as wound healing and drug delivery,^[^ ^42^ ^]^ recent studies suggest promising potential for their use in environmental applications, including pollutant removal.^[^ ^43^ ^]^ These advancements open up new possibilities for leveraging algal nanofibers in both environmentally conscious and impactful ways.

In accordance with these advancements, the present study adopted a different approach, and Ag nanoparticles were synthesized using the powders of algae (*Cystoseira barbata* and *Spirulina* sp.) along with nanofiber structures. Beyond conventional biosynthesis techniques, nanofibers containing *Cystoseira barbata* (PAN/*Cb*) facilitated the homogeneous distribution of nanoparticles, leading to more uniform and controlled sizes of silver nanoparticles. Additionally, the use of xanthan gum (XG) significantly enhanced nanoparticle stability, addressing a common challenge in biosynthesis. The antimicrobial properties of the obtained nanoparticles were thoroughly investigated. Furthermore, the reusability of the reducing agent used for the first time in the biosynthesis process (PAN/*Cb*/Ag@XG) for photocatalytic applications was aimed, at ensuring an environmentally friendly approach and sustainable use potential. This innovative approach not only contributes to the biosynthesis of silver nanoparticles but also demonstrates a circular, sustainable method for water treatment and disinfection, highlighting both environmental responsibility and long‐term effectiveness.

## 2 Results and Discussion

Traditionally, toxic reducing agents such as sodium borohydride (NaBH_4_), ninhydrin (C_9_H_6_O_4_), and citrate salts have been commonly employed for metal nanoparticle synthesis. However, in recent years, there has been growing interest in utilizing natural biological sources for nanoparticle synthesis due to their eco‐friendly nature. Notably, we explored the efficacy of both powdered and fiber forms of *Spirulina* sp. and *C. barbata* as reducing agents in synthesizing Ag nanoparticles, offering novel insights into the potential of algal materials for nanoparticle production.

### 2.1 Characterization and Optimization of Ag Nanoparticle Synthesis Using Algae Powder and Algal Nanofibers

**Figure 1:**
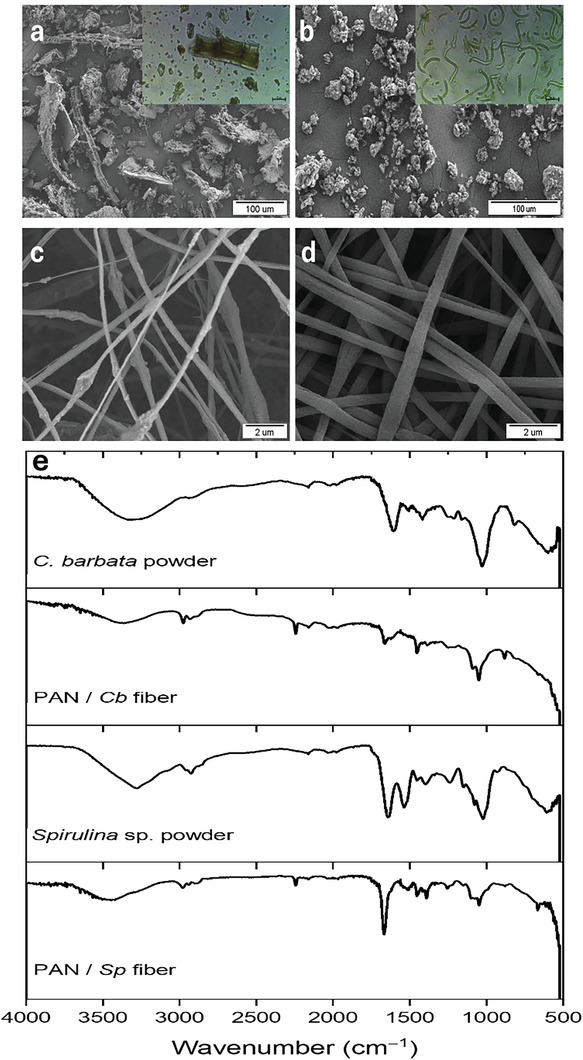
SEM micrographs of powdered *C. barbata* a) *Spirulina* sp. b), (Insets show optical microscopy images of the powder), PAN / *Cb* nanofiber c) PAN / *Sp* nanofiber d), FTIR spectra of the employed reducing agents e).

The SEM micrographs of the reducing agents in powder and nanofiber forms are depicted in **Figure** **1**a–d, accompanied by optical microscopy images inset. Notably, the morphological structure of *Spirulina sp*. powder differs significantly from that of *C. barbata* powder. *C. barbata* exhibits a non‐homogeneous, porous, and fibrous structure, indicating its potential for providing a larger surface area. The optical microscope images also provide insights into the characteristics of these algae. *Spirulina* derives its name from its spiral or helical filament arrangement, with its green color attributed to chlorophyll pigments. The filament lengths within this microscopic threadlike structure range from 50 to 250 µm, while the diameter of the cylindrical cells (trichomes) is approximately 10 µm. Conversely, *C. barbata*, a perennial brown algae, features a round or flat woody structure comprising various cell types and tissues. Its thick cell walls primarily consist of components like cellulose and alginate. In the provided image, the thallus length measures 230 µm, with a diameter of approximately 50 µm. The observation that the diameter of PAN/*Cb* nanofibers (284 ± 80 nm) is smaller than that of PAN/*Sp* nanofibers (373 ± 117 nm) suggests that PAN/*Cb* nanofibers possess an even larger surface area. The diameter distributions and physical appearances of PAN/*Cb* and PAN/*Sp* nanofibers are demonstrated in Figure S1 (Supporting Information).

Figure 1e shows the FTIR spectra, which were performed to identify the bioactive compounds present in powdered algae and algal nanofibers and to determine their roles in the reduction of Ag^+^ ions. The band observed at 2971 cm⁻¹ in both *C. barbata* powder and PAN/*Cb* nanofiber samples can be attributed to C─H stretching.^[^ ^44^ ^]^ In the *C. barbata* spectrum, the band at 1628 cm⁻¹ represents C═O stretching, while the bands observed at 1402, 1039, and 878 cm⁻¹ are associated with O─H bending, C─O stretching, and ─S═O stretching, respectively.^[^ ^43^, ^45^ ^]^ In the PAN/*Cb* nanofiber spectrum, the band at around 2240 cm^−1^ is attributed to ─C≡N nitrile groups of PAN.^[^ ^46^ ^]^ Moreover, the bands at 1422 and 1446 cm⁻¹ correspond to O─H bending in carboxylic acid and alcohol,^[^ ^47^ ^]^ while the C═O band at 1656 cm⁻¹, the C–O band at 1059 cm⁻¹, and the ─S═O stretching band at 873 cm⁻¹ are attributed to the contributions from *C. barbata*.^[^ ^48^ ^]^ The characteristic bands of *Spirulina* powder, primarily attributed to its main component, protein, exhibit strong amide I (C═O stretching) at 1640 cm⁻¹ and amide II at 1541 cm⁻¹. Additionally, saccharides in *Spirulina* contribute bands in the range of 1160–1000 cm⁻¹, corresponding to C─O or C─C vibrations.^[^ ^49^, ^50^ ^]^ Similarly, the FTIR spectrum of PAN/*Sp* nanofibers shows distinct bands from both PAN and *Spirulina*, with noticeable shifts in the amide and nitrile regions. These shifts indicate interactions between the components, confirming the successful incorporation of *Spirulina* into the PAN nanofiber matrix.

**Figure 2:**
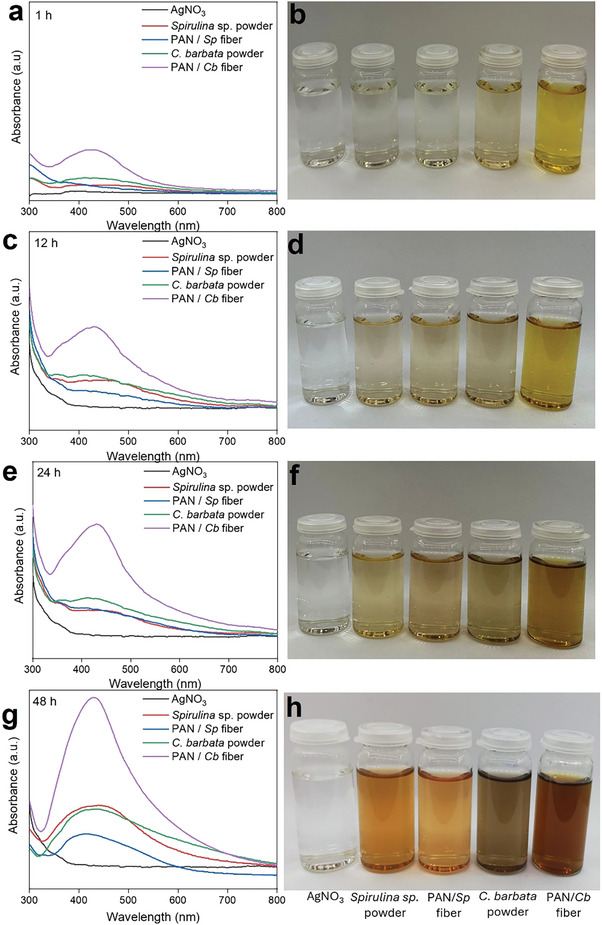
UV–vis spectra and corresponding photographs depict the color evolution during the formation of Ag nanoparticles from a 3 mM AgNO_3_ solution (20 mL) using various reducing agents (2 mg powder or 20 mg fiber) over different times: a, b) 1 hour, c, d) 12 hours, e, f) 24 hours, and g, h) 48 hours.

UV‐visible spectra and color changes of Ag nanoparticles obtained from AgNO_3_ solution with different reducing agents (*Spirulina* sp. and *C. barbata* in powder and nanofiber form) and different times (1, 12, 24, 48 hours) are demonstrated in **Figure** **2**. While a strong surface plasmon resonance (SPR) at approximately 427 nm proves the formation of Ag nanoparticles, the increase in intensity with time may be due to the increase in the number of nanoparticles formed as a result of the decrease in Ag^+^ ions present in the aqueous solution. The highest absorbance was obtained in the presence of *C. barbata* fiber at 48 hours. However, while the maximum wavelength was 427 nm after 1 hour, a shift to 432 nm was observed with increasing time. The shift of absorbance to a longer wavelength region (red shift) is associated with the increase in Ag nanoparticle size.^[^ ^51^ ^]^ As the size increases, their intensity increases due to the larger reactive surface area.

After adding *Spirulina* sp. powder and nanofibers, the resulting Ag nanoparticle solutions changed from colorless to yellowish‐orange, while after adding *C. barbata* powder and nanofibers, they turned yellowish‐brown. The color difference is believed to be due to the algae's varying flavonoid and phenolic acid content. Generally, algae are classified as green (chlorophytes), red (rhodophytes), and brown (phaeophytes) based on their body or thallus pigmentation. Brown algae are known to contain significant amounts of phenolic compounds and various natural functional components (terpenes, alkaloids, and amino and fatty acids).^[^ ^52^, ^53^ ^]^

**Scheme 1:**
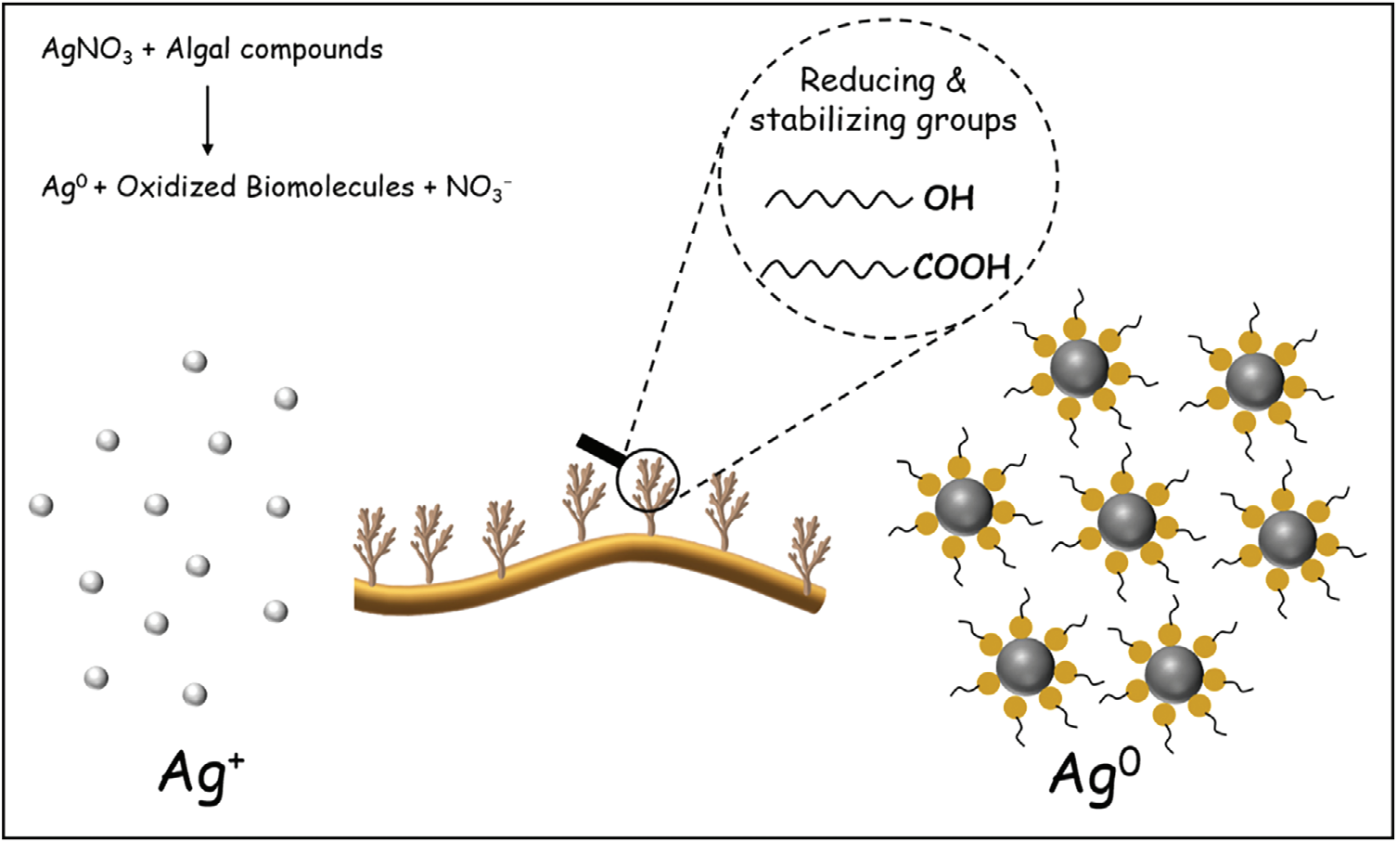
A schematic representation of algal‐mediated Ag nanoparticle formation.

**Scheme** **1** demonstrates the tentative mechanism for Ag nanoparticle formation in the presence of brown algae acting as both reducing and capping agents. Initially, AgNO_3_ dissolves in water, releasing Ag⁺ ions. Algal compounds such as phenolics, flavonoids, polysaccharides (fucoidan, alginate), and proteins donate electrons, reducing Ag⁺ to metallic Ag^0^ atoms that nucleate and grow into nanoparticles under controlled conditions.^[^ ^37^ ^]^ Meanwhile, polysaccharides and proteins in the algal compounds cap the nanoparticles, stabilizing them by preventing excessive aggregation through steric hindrance and electrostatic repulsion, ensuring the formation of well‐dispersed colloidal Ag nanoparticles.

Aboelfetoh et al. synthesized Ag nanoparticles using the aqueous extract of *Caulerpa serrulata* (green marine macroalgae) from AgNO_3_ solution, with the color shifting from light yellow to reddish‐brown.^[^ ^20^ ^]^ Similarly, the liquid extract of the cyanobacterium *Oscillatoria limnetica* changed from green to brown when treated with the same precursor, which was linked to the biotransformation of Ag^+^ ions to Ag^0^.^[^ ^54^ ^]^ In another study, the controlled addition of a liquid extract of *Portieria hornemannii* to AgNO_3_ solution resulted in a color change from light pink to dark brown.^[^ ^32^ ^]^ In addition to the type of reducing agent, morphology can affect the reduction and nucleation rates. In this study, nanofiber‐based reducing agents (PAN/*Sp* and PAN/*Cb*) produced more Ag nanoparticles than the powder‐based agents (*Spirulina* sp. and *C. barbata*), likely due to the greater accessibility of algae in the nanofiber form.

Another study compared the biosynthesis of Ag nanoparticles using cinnamon bark powder and liquid extract. After adding the powder‐based agent to the AgNO_3_ solution, a noticeable color change from colorless to pale yellow occurred within two hours. In contrast, the liquid extract, initially pale reddish‐brown, darkened upon addition to the precursor solution, accompanied by an increase in absorbance. This was attributed to the higher concentration of reducing agents in the liquid extract.^[^ ^55^ ^]^

**Figure 3:**
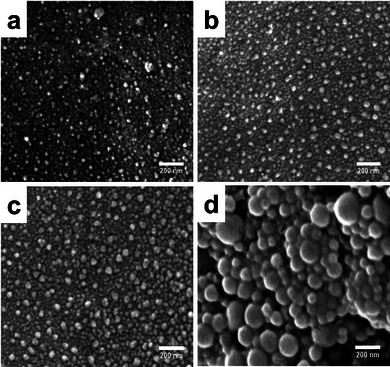
SEM micrographs of Ag nanoparticles obtained from the treatment of powdered *Spirulina* sp. a) and *C. barbata* b), and nanofibers of PAN/*Sp* c) and PAN/*Cb* d) with AgNO₃ solution (20 mL, 3 mM) for 24 hours.

The morphologies of Ag nanoparticles obtained from the treatment of reducing agents in both powder and nanofiber forms are shown in **Figure** **3**. The heterogeneous, porous, and fibrous structure of *C. barbata* (Figure 1a) may offer a larger surface area. Although the size of Ag nanoparticles formed from algae powders is smaller than those from algal nanofibers, with their typically larger surface area, facilitate more controlled and efficient nanoparticle formation, providing more opportunities for metal ion reduction. Spherical‐shaped Ag nanoparticles were obtained from the treatment of *Spirulina* sp. powder (Figure 3a) and PAN/*Sp* nanofibers (Figure 3c). Similarly shaped Ag nanoparticles with wider distribution, formed from *C. barbata* powder (Figure 3b) and PAN/*Cb* nanofibers (Figure 3d). The diameter distributions of Ag nanoparticles from different reducing agents are shown in Figure S2 (Supporting Information). Based on these findings, PAN/*Cb* nanofibers were selected for further studies due to the higher rate of nanoparticle formation observed. The smaller diameter of PAN/*Cb* nanofibers, compared to PAN/*Sp* nanofibers, is suggested to provide an even larger surface area, further enhancing their potential for efficient and uniform Ag nanoparticle synthesis. (see also Figure 2).

**Figure 4:**
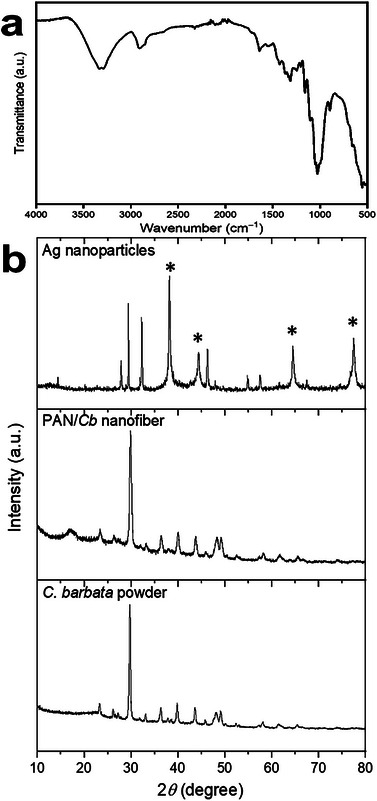
FTIR spectrum a) and XRD pattern b) of Ag nanoparticles obtained from treating PAN/*Cb* nanofiber (20 mg) with AgNO₃ solution (20 mL, 3 mM) for 24 hours. For comparison, the XRD patterns of PAN/*Cb* nanofiber and *C. barbata* powder are also presented.

**Figure** **4**a shows the FTIR spectrum of Ag nanoparticles synthesized from PAN/*Cb* nanofiber reveals key functional groups involved in their formation and stabilization. A broad band around 3400 cm⁻¹ corresponds to O‐H stretching vibrations from water molecules or alcohol groups in the nanofiber, which likely aid in the reduction of Ag^+^ ions. The bands around 500–600 cm⁻¹ is associated with the phenolic compounds^[^ ^56^ ^]^ or phytoconstituents of *Cb*.^[^ ^53^ ^]^

XRD pattern of Ag nanoparticles in Figure 4b shows the diffraction signals at 38.3°, 44.3°, 64.5°, and 77.5° 2θ, which align with the (111), (200), (220), and (311) Bragg reflections, indicating that the synthesized nanoparticles have a crystalline and face‐centered cubic structure.^[^ ^47^ ^]^ The diffraction signals observed at 23.3° in the patterns of PAN/*Cb* nanofibers and *C. barbata* powder correspond to the presence of alginate from the brown algae.^[^ ^57^ ^]^ Other diffraction signals associated with 87% magnesium calcite and 13% aragonite, which originate from the mineral structure of *C. barbata*.^[^ ^58^ ^]^ Unlike *C. barbata* powder, the diffraction pattern of PAN/*Cb* nanofiber shows a characteristic signal at 17.1°, which corresponds to the orthorhombic packing of PAN, resulting from the stretching of chains during the electrospinning.^[^ ^59^ ^]^

**Figure 5:**
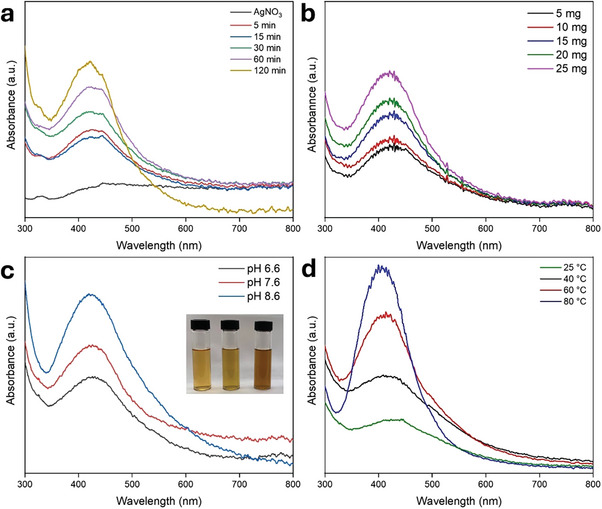
The optimization of Ag NP synthesis using PAN/*Cb* nanofiber‐AgNO_3_ (3 mM, 20 mL) under different parameters: a) time duration (20 mg nanofiber, pH 6.6, 25 °C), b) amount of PAN/*Cb* nanofiber (120 min, pH 6.6, 25 °C), c) pH (25 mg nanofiber, 120 min, 25 °C), and d) temperature (25 mg nanofiber, 120 min, pH 8.6).

**Figure** **5** illustrates the optimization of Ag nanoparticle synthesis using PAN/*Cb* nanofibers under varying parameters. An increase in contact time led to a rise in the intensity, indicating continued reduction of Ag⁺ ions and an increasing concentration of Ag nanoparticles. A pronounced band at 422 nm confirms the successful formation of Ag nanoparticles. Based on these observations, a contact time of 2 hours was determined to be suitable for the efficient synthesis of Ag nanoparticles under the specified conditions (Figure 5a). Increasing the amount of PAN/*Cb* nanofibers from 5 mg to 25 mg led to a significant enhancement of the band (Figure 5b). In the presence of PAN/*Cb* nanofibers, the initial pH of the AgNO₃ solution was measured to be 6.6. Ag nanoparticle formation was then monitored at pH values of 7.6 and 8.6. The results suggest that the reduction capability of PAN/*Cb* nanofibers improves under alkaline conditions. As the pH increased, both absorbance and the width of the band increased, with a shift to a lower wavelength (417 nm). This broadening and blue shift indicate the formation of smaller Ag nanoparticles in greater quantities, accompanied by a tendency for aggregation (Figure 5c). Temperature also plays a crucial role in the synthesis of Ag nanoparticles, as higher temperatures increase the reaction rate due to the faster consumption of reactants, leading to the formation of smaller nanoparticles.^[^ ^60^ ^]^ As the temperature increased from 25 °C to 80 °C, the less intense band became more pronounced (Figure 5d). The optimum parameters for the efficient synthesis of Ag nanoparticles were determined as a contact time of 2 hours, 20 mg of PAN/*Cb* nanofibers, a pH of 8.6, and room temperature, ensuring efficiency, cost‐effectiveness, and energy conservation.

### 2.2 Stability, Reusability, and Morphological Analysis of Ag Nanoparticles Synthesized with PAN/*Cb* Fibers

**Figure 6:**
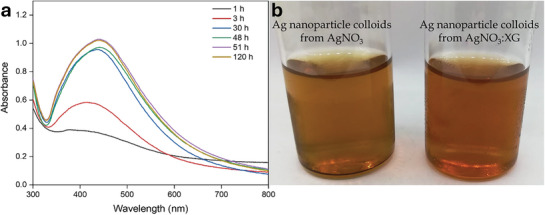
UV–vis spectrum showing the time‐dependent evolution of Ag nanoparticles obtained from treating a solution of AgNO_3_:XG (1:1, 3 mM, 20 mL) with PAN/*Cb* fiber (20 mg) a), accompanied by a photographic image showing the initially formed Ag nanoparticles and their state after 3 months b).

Stability is crucial in the synthesis and storage of nanoparticles because insufficient stability in colloidal nanoparticles can lead to limitations in applications involving nanomaterials. To ensure stability and achieve a homogeneous dispersion, xanthan gum (XG), rich in carboxylic acid groups, was added to the precursor solution. **Figure** **6**a shows the stability of Ag nanoparticles synthesized in the presence of XG at different time intervals. The results indicated that, although slight changes were observed in the UV‐vis spectra after 6 months, narrow and sharp absorption bands, indicating the presence of stable colloidal Ag nanoparticles, were still present. The photographs of Ag nanoparticle solutions synthesized with and without XG after 3 months are depicted in Figure 6b. In the absence of XG, Ag nanoparticles settled at the bottom of the solution. However, when XG was present, the stability of the Ag nanoparticles improved, effectively preventing aggregation. The carboxyl groups, derived from glucuronic and pyruvic acids in XG, are highly electronegative and play a pivotal role in stabilizing the nanoparticles. These groups induce electrostatic repulsion between the Ag nanoparticles, keeping them dispersed in water and ensuring their long‐term stability.^[^ ^61^, ^62^ ^]^

**Figure 7:**
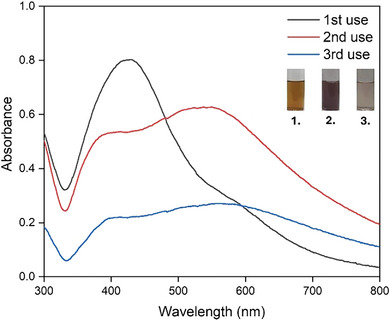
UV–vis spectra and color transformations during the formation of Ag nanoparticles achieved through successive usage of PAN / *Cb* fiber (20 mg) with AgNO_3_:XG (1:1, 3 mM, 20 mL, 12 h) solution.

The reduction ability of PAN/*Cb* nanofibers was evaluated through reusability studies. After the fibers were reduced and dried, they were exposed to fresh metal solutions for further reactions. The UV–visible spectra of Ag nanoparticle formation and the photographs showing color changes are presented in **Figure** **7**. In the second and third uses of the PAN/*Cb* nanofiber, the absorption peak shifted to a longer wavelength, indicating an increase in the size of the Ag nanoparticles. Additionally, a secondary peak at a lower wavelength, resulting from quadrupole resonance in addition to the primary dipole resonance, became more pronounced.^[^ ^63^ ^]^

**Figure 8:**
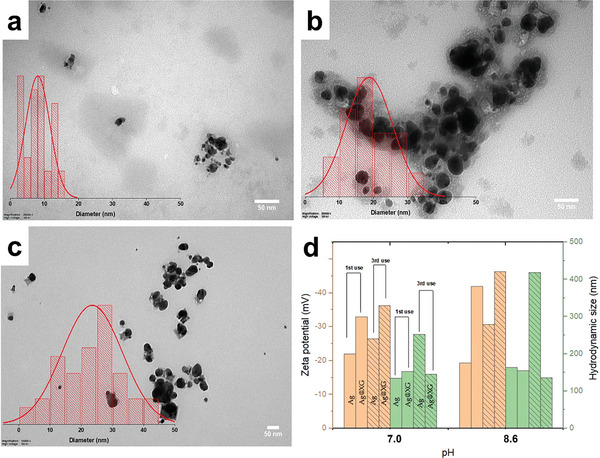
TEM micrographs and diameter distributions of Ag nanoparticles after successive usage (1st, 2nd, and 3rd use from a to c) of PAN/*Cb* fibers (20 mg) with AgNO₃:XG (1:1, 3 mM, 20 mL, 12 h) solution, size distributions and zeta potentials of Ag nanoparticles from the 1st and 3rd use (d).

**Figure** **8**a–c show the TEM micrographs revealing a progressive increase in Ag nanoparticle size with successive usage. The nanoparticle diameters increased by more than 2‐fold (from 8.04 ± 3.52 nm to 18.74 ± 6.75 nm) after the second cycle, with significant agglomeration observed, evidenced by the formation of larger clusters. This agglomeration resulted in a pronounced color darkening of the composite, attributed to changes in the SPR. After the third cycle, nanoparticles were predominantly deposited on the fiber surface, leading to a pale coloration, likely due to the reduction in suspended nanoparticles (23.52 ± 9.58 nm). These findings were corroborated by the red shift observed in the UV–vis spectra in Figure 7, indicative of particle growth and agglomeration. Figure 8d illustrates the relationship between the zeta potential and the hydrodynamic radius of Ag nanoparticles under varying pH conditions. The stability of Ag nanoparticles was assessed at pH 7 and pH 8.6, where zeta potentials were measured as −32.9 mV and −41.9 mV, respectively, indicating good colloidal stability. Correspondingly, hydrodynamic diameters were 153 nm (PDI = 0.324) and 155 nm (PDI = 0.387), showing minimal change, suggesting stable particle sizes across the tested pH range. After the third usage, both zeta potential and hydrodynamic size exhibited negligible changes, further confirming that the particles maintained their charge and size stability. This observation implies robust electrostatic stabilization under the given conditions. In the presence of XG, silver nanoparticles synthesized under identical conditions displayed higher zeta potential values. This can be attributed to the carboxylic acid groups in XG, which, at elevated pH, deprotonate to form carboxylate ions. The increased negative charge enhances electrostatic repulsion, effectively preventing agglomeration and yielding a more stable colloidal system. The results underline the role of XG in promoting stability by increasing negative surface charge and steric hindrance. Besides, XG enhances nanoparticle stability and reusability by acting as a capping agent, preventing aggregation and extending shelf life. For example, the zeta potential of N@NiHCF nanomaterials improved from −17.7 mV to −22.9 mV in the XG‐based GGXa@N@NiHCF nanocomposite, indicating reduced flocculation and improved structural stability.^[^ ^64^ ^]^ Similarly, XG‐capped Cr₂O₃ nanoparticles by blocking active sites prone to aggregation, significantly increasing their shelf life compared to bare nanoparticles.^[^ ^65^ ^]^ Therefore, polysaccharides accelerate nanoparticle formation, as seen in Ag nanoparticle systems,^[^ ^66^, ^67^ ^]^ emphasizing their critical role in enhancing stability, controlling nucleation, and supporting diverse applications in nanotechnology.

### 2.3 Antimicrobial Properties of Ag nanoparticles

**Figure 9:**
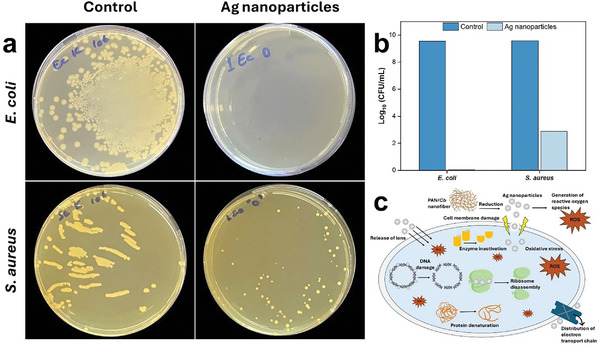
Antibacterial activity of Ag nanoparticles against *E. coli* and *S. aureus*: The representative images of agar plates showing the bacterial colonies in control (untreated) and Ag nanoparticle‐treated samples a), quantitative analysis of bacterial growth b), schematic illustration of the antibacterial mechanism of Ag nanoparticles c).

The bacterial inhibition activity of Ag nanoparticles was investigated against *S. aureus* and *E. coli* (**Figure** **9**). Biogenically synthesized Ag nanoparticles derived from PAN/*Cb* nanofibers were found to effectively reduce the number of viable colonies (Figure 9a). Ag nanoparticles showed high antibacterial activity (approximately 100%) against *E. coli*, and also exhibited a significant effect against *S. aureus* (Figure 9b), suggesting higher efficacy against Gram‐negative bacteria. This difference likely stems from structural variations in bacterial cell walls, as the thinner peptidoglycan layer greater biofilm formation in Gram‐negative bacteria allows easier nanoparticle penetration and disruption.^[^ ^68^, ^69^ ^]^ In contrast, the thicker cell wall of *S. aureus* limits nanoparticle penetration, despite its susceptibility to Ag⁺ ions once inside. These structural and biofilm properties explain the stronger antibacterial effects observed against *E. coli*.

Figure 9c shows the antibacterial mechanism of Ag nanoparticles involves a series of interactions with bacterial cells, and these interactions affect the integrity of cellular structures and metabolic processes. Initially, Ag nanoparticles interact with the bacterial cell surface through electrostatic attraction to bacterial membrane components such as lipopolysaccharides in Gram‐negative bacteria due to their negative charge. This physical interaction allows the nanoparticles to adhere to the cell and facilitates the penetration of Ag⁺ ions into the cell membrane. These ions are highly reactive and bind to bacterial DNA, inhibiting replication and transcription. Additionally, Ag⁺ ions disrupt essential enzymes, leading to metabolic dysfunction. Ag nanoparticles can also damage the bacterial cell membrane, increasing its permeability and causing leakage of cellular contents, leading to cell death. Another important mechanism is the production of ROS, which induces oxidative stress and damages cellular components such as lipids, proteins, and DNA. Moreover, Ag nanoparticles can disrupt bacterial biofilms, making bacteria more susceptible to antimicrobial agents. These combined interactions—membrane damage, DNA damage, enzyme inhibition, ROS production, and biofilm disruption—result in Ag nanoparticles exhibiting strong antibacterial activity against a wide range of bacteria.^[^ ^70^, ^71^ ^]^

The dual functionality of Ag nanoparticles lies in their ability to target and eliminate harmful pathogens while minimizing the need for harsh chemical disinfectants that can lead to secondary pollution. This makes them an ideal candidate for advanced wastewater treatment systems, addressing both public health concerns and the growing demand for eco‐friendly water purification technologies.

### 2.4 Photocatalytic Performance, Reusability, and Ag Nanoparticle Efficiency of PAN/*Cb*/Ag@XG Nanofibers

**Figure 10:**
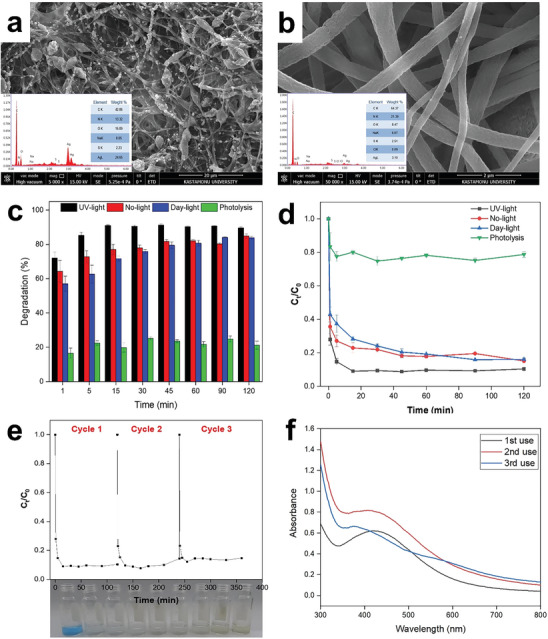
SEM micrographs and EDX spectra of the PAN/*Cb*/Ag@XG nanofibers before a) and after b) MB degradation, degradation efficiency of PAN/*Cb*/Ag@XG nanofibers c), normalized concentration with time d) under different conditions, and reusability test results after successive usage of PAN/*Cb*/Ag@XG nanofibers along with the photographic images of MB after 3rd cycle e), and Ag nanoparticle production efficiency of PAN/*Cb* nanofibers for each cycles (f).

**Figure** **10**a presents the SEM micrograph of PAN/*Cb*/Ag@XG nanofibers, showing a uniform distribution of Ag nanoparticles, clearly visible as bright spots on the nanofiber surface. EDX spectrum confirms the presence of Ag, indicating successful functionalization. After MB degradation, the micrograph in Figure 10b reveal the removal of Ag nanoparticles from the fiber surface, while the nanofiber structure remains intact, demonstrating excellent mechanical stability. EDX spectrum of post‐degradation show a diminished Ag signal, confirming nanoparticle involvement in the photocatalytic process. The preserved integrity of the nanofibers suggests they can be re‐functionalized with Ag nanoparticles, enabling their reuse as a reducing agent and subsequently as a photocatalyst. The photocatalytic activity of PAN/*Cb*/Ag@XG nanofibers was determined using MB under no‐light, day‐ and UV‐light irradiation. Organic molecules degrade in the presence of a photocatalyst through H⁺ ions and hydroxyl radicals (OH•) generated by electron pairs (e⁻) and holes (h⁺).^[^ ^72^ ^]^ The photocatalytic efficiency of PAN/*Cb*/Ag@XG nanofibers in MB degradation was evaluated by monitoring the time‐dependent decrease in MB's absorbance at 664 nm. According to these results, the degradation of MB occurred rapidly within the first minute of light exposure, the intensity of the absorption band decreased, and the color degradation reached equilibrium after 45 minutes under UV light, while the time to reach equilibrium under daylight increased to 120 minutes. At 45 minutes, while the degradation percentage in the absence of the fiber catalyst was 23.61 ± 0.10%, it was determined as 81.76 ± 1.12%, 79.54 ± 1.88%, and 91.23 ± 0.76 in the presence of PAN/*Cb*/Ag@XG nanofibers under no‐light, day‐ and UV‐light conditions, respectively (Figure 10c).

The decrease in absorbance is attributed to the critical role of PAN/*Cb*/Ag@XG nanofibers as catalysts in the degradation of MB dye. However, in a system containing only the dye, no significant decrease in the absorbance spectra was observed even after 120 minutes, indicating the degradation can be effectively achieved without the need for a reducing agent such as NaBH_4_. The photocatalytic activity of nanoparticles is associated with electron donation and acceptance, a mechanism that can be explained by their ability to act as a substrate for electron transfer on their surface.^[^ ^73^ ^]^ The photocatalytic properties of PAN/*Cb*/Ag@XG nanofibers can be triggered by the excitation of SPR and charge density oscillations may be responsible for the loading at the interface between the phase and dielectric media.^[^ ^74^ ^]^ Therefore, Ag nanoparticles on the nanofibers function as electron transfer channels, altering the chemical properties of the dye and neutralize its harmful effects.

The rate constants of the MB degradation reactions for PAN/*Cb*/Ag@XG nanofibers were determined from the slopes of the –*ln*(C*t*/C_0_) versus time curves plotted from Figure 10d. The calibration plots under various conditions is shown in Figure S3 (Supporting Information). The degradation reaction followed pseudo‐first‐order reaction kinetics, and the corresponding rate constant k values under no‐light, day‐ and UV‐light irradiation were found to be 9.8 × 10^−3^ min⁻¹, 1.18 × 10⁻^2^ min⁻¹, and 2.49 × 10⁻^2^ min⁻¹, respectively. The increased dye degradation rate of PAN/*Cb*/Ag@XG nanofibers under UV light compared to daylight can be attributed to several key factors. UV light has shorter wavelengths and higher energy than visible light, enabling more effective excitation of electrons in nanoparticles, leading to the generation of electron‐hole pairs that initiate dye degradation. UV light also enhances SPR in Ag nanoparticles, resulting in the generation of ROS, such as hydroxyl radicals and superoxide anions, which play a crucial role in breaking down dye molecules. For practical use, maintaining photostability and ensuring recyclability are significant as achieving high photocatalytic efficiency.^[^ ^75^ ^]^ Figure 10e demonstrates that PAN/*Cb*/Ag@XG nanofibers effectively degrade MB dye over successive cycles under UV‐light, with photocatalytic degradation efficicency ranging from 91.25% to 85.48% over three cycles. Interestingly, the nanoparticle production efficiency of the used PAN/*Cb* nanofibers remains stable even after degradation, which is shown in Figure 10f.

Recently, many studies have reported that Ag nanoparticles obtained through green synthesis provide effective photocatalytic activity. **Table** **1** lists various studies on Ag nanoparticles synthesized using different reducing agents and photocatalytic conditions. The reducing agents range from plant extracts (e.g., Ficus altissima, Jasmine flower, Pistachio husk) to biomaterials like algae (*Chlorella sorokiniana, Padina pavonica*). The size of Ag nanoparticles varies widely (0.6 nm to 79 nm) and are characterized using techniques such as TEM, DLS, and SEM. Photocatalytic dye degradation efficiencies depend on factors like co‐catalysts (e.g., NaBH₄), light sources (UV light, sunlight), and reaction times, with degradation percentages reaching up to 100% in many cases. The current study, employing PAN/*Cb*/Ag@XG nanofibers, demonstrates high photocatalytic efficiency (91.23% in 45 minutes), comparable or superior to other studies, particularly in terms of degradation rate and nanoparticle size uniformity.

**Table 1:** Summary of studies on the green synthesis and application of Ag nanoparticles for photocatalytic dye degradation.

Nano‐particles/composites	Reducing agent	Ag NPs size/Technique	Dye	Co‐catalyst	Light Source	Time/Percentage	References
Ag/PVA nanofibers	*Ficus altissima Blume* extract	4.9 nm/TEM	MB (20 mg L^−1^)	KBH_4_	UV‐light	15 min; 100%	[76]
Ag nanoparticles	*Gmelina arborea extract*	17 nm/TEM	MB (10 mM)	NaBH_4_	–	10 min; 100%	[77]
Ag nanoparticles	*Euphorbia geniculata* leaf extract	8 to 19 nm (XRD and SEM)	MO (25 mg L^−1^)	NaBH_4_	–	30 min; 97.28%	[78]
Ag nanoparticles	*Jasmine* flower extract	10 nm to 40 nm/SEM	MB	–	Sun light	120 min; 72%	[79]
Ag nanoparticles	*Mangifera indica gum* extract	19 nm/ TEM	MB (10 mg L^−1^)	–	UV‐light	75 min; 94%	[80]
Cellulose based silver nanocomposite	*Laurus nobilis extract*	25 nm/TEM	MB (10 mg L^−1^)	NaBH_4_	–	120 min; 100%	[81]
Ag nanoparticles	*Chlorella sorokiniana* extract	79 nm (DLS)	MB (5 mg L^−1^)	–	Sun light	18 hours; 95.75%	[82]
Ag nanoparticles	*Sargassum horneri* extract	90.8 nm (DLS) 22.72 nm (TEM)	MB (0.08 mM), MO (0.10 mM), RB (0.05 mM)	NaBH_4_	–	12 min; ‐ 22 min; ‐ 15 min; ‐	[83]
Ag nanoparticles	*P. hortorum* flower *Allium fistulosum* stem (Afs)	2 nm (AgNPs/Phf) 0.6 nm (AgNPs/Afs) /TEM	Blue sky No. 39 and Green emerald No. 19 (0.0374 g L^−1^).	–	Sun light	AgNPs/Phf 240 min;95% AgNPs/Afs 5 min:100%	[84]
Ag nanoparticles@cellulose nanocomposites	Pistachio husk	24 and 25 nm/TEM	Direct Blue 151 (5.3 × 10^−3^ M)	NaBH_4_	Natural sun light	instantaneously; 100%	[85]
AgNPs‐PA66 nanofiber	*Helichrysum arenarium* extract	28 nm/TEM	MO (10 mg L^−1^)	NaBH_4_	UV‐light	30 min; 100%	[86]
PPAg‐NPs	*Padina pavonica* extract	30 nm to 90 nm/DLS	MB	–	UV‐light	105 min; 90%	[75]
PAN/*Cb*/Ag@XG nanofibers	PAN*/Cb* nanofiber	8.04 nm/TEM	MB (5 mg L^−1^)	–	UV‐light	45 min; 91.23%	**This study**

While most studies rely on plant‐based reducing agents and co‐catalysts (e.g., NaBH₄), often involving extraction steps, the current study takes a unique approach using PAN/*Cb* nanofibers as a reducing mat, which eliminates the need for co‐catalysts and avoids handling powdered or liquid substances. Additionally, after reduction, PAN/*Cb* nanofibers were successfully decorated with Ag nanoparticles (PAN/*Cb*/Ag@XG), creating a reusable photocatalytic system (see also Figure 10f). These nanofibers can be effectively applied for photocatalytic degradation, offering the advantage of multiple uses without compromising performance, making them a sustainable and practical alternative for dye removal applications.

## 3 Conclusion

The present study pioneers an innovative approach for the biosynthesis of Ag nanoparticles using algae powders (*C. barbata* and *Spirulina* sp.) and algal nanofibers (PAN/*Cb* and PAN/*Sp*). Among these, PAN/*Cb* nanofibers proved to be the most effective reducing agent, enabling a higher rate of Ag nanoparticle formation while ensuring their homogeneous distribution and producing uniform, controlled sizes of Ag nanoparticles. The incorporation of xanthan gum (XG) significantly enhanced the stability of the nanoparticles, preventing aggregation through electrostatic repulsion induced by the carboxyl groups in XG. This ensured the long‐term stability and dispersion of Ag nanoparticles in water. The synthesized Ag nanoparticles demonstrated remarkable antibacterial activity against *E. coli* and *S. aureus*. After the reduction process, the resulting material, PAN/*Cb*/Ag@XG nanofibers, was optimized for methylene blue (MB) degradation under various light conditions. Ag nanoparticles acted as electron transfer channels, effectively neutralizing dye contaminants with degradation efficiencies of 81.76%, 79.54%, and 91.23% under no‐light, daylight, and UV‐light conditions, respectively, surpassing the performance without a catalyst.

This study not only advances the biosynthesis of silver nanoparticles but also aligns with principles of environmental responsibility and sustainability. The findings pave the way for practical, cost‐effective, and energy‐efficient solutions in water treatment and public health, addressing the critical demand for eco‐friendly technologies.

## 4 Experimental Section

### Materials

Polyacrylonitrile (PAN, Mw = 150000 g mol^−1^), *N, N*‐dimethylformamide (DMF) and nitric acid (HNO_3_) were acquired from Sigma Aldrich. Silver nitrate (AgNO_3_) and sodium hydroxide (NaOH) were obtained from Merck. The brown macroalgae (*C. barbata*) was collected from the coast of Hamsilos, located in Sinop, Türkiye, and the microalgae (*Spirulina* sp., 100% Arthrospira) was purchased from İskoç Organik (Seydikemer, Muğla). The *C. barbata* samples were washed with distilled water to remove impurities. After washing, they were dried in a lyophilizer at −40 °C and 1 atm pressure. The specific lyophilizer model used was XQ‐Instrument freeze dry/XQ‐12B, as mentioned in the reference. Both algae samples were separately fractionated into smaller pieces with a disc mill (Retsch RS 200) and subsequently sieving them using a mesh sieve with approximately 100 µm openings. After fractionation, the sieved algae samples were stored in a refrigerator at 4 °C for future use. Ultrapure water sourced from the Milli‐Q System by Millipore was utilized throughout the experiments.

### Fabrication of PAN/Cb and PAN/Sp Nanofibers

PAN (10 wt%) was dissolved in DMF at room temperature and stirred in a magnetic stirrer (Velp Stirring Line) at 300 rpm for 24 h to form a homogeneous solution. The powdered *C. barbata* or *Spirulina* sp., at a final concentration of 10 wt%, was dispersed in DMF for 1 h in an ultrasonic bath. These two solutions were mixed together using a magnetic stirrer for a duration of 5 hours. PAN/*Cb* and PAN/*Sp* solutions were transferred into 10 mL plastic syringes. The plastic syringe was placed horizontally on the micro syringe pump of the electrospinning device (Inovenso Basic Set‐up). A voltage of 14 kV was applied to the solutions at room temperature at a flow rate of 1 mL h^−1^ with a tip collector distance of 19 cm and the nanofibers were collected on an aluminum foil collector.^[^ ^43^ ^]^ The electrospinning time was kept constant at 2 hours.

### Biogenic Synthesis of Silver Nanoparticles

*C. barbata* and *Spirulina* sp. powders as well as PAN/*Cb* and PAN/*Sp* nanofibers were utilized as reducing agents with AgNO_3_. First, aqueous stock solutions of AgNO_3_ at a concentration of 3 mM were prepared. *C. barbata* or *Spirulina* sp. powders (2 mg), PAN/*Cb* or PAN/*Sp* nanofibers (20 mg) were immersed separately in solutions containing AgNO_3_ (3 mM, 20 mL). The solutions were stirred in a magnetic stirrer at room temperature for different times (1‐12‐24‐48 h) and then filtered and absorbance values were measured by UV–Vis spectrophotometer. Additionally, AgNO_3_ precursor solution (3 mM) and PAN/*Cb* nanofibers (20 mg) were selected as reducing agents, exploring the impacts of varying parameters such as time (5‐15‐30‐60‐120 min), amount of reducing agents (5‐10‐15‐20‐25 mg), pH levels (6.6‐7.6‐8.6), and temperature (25‐40‐60‐80 °C) on the synthesis of Ag nanoparticles.

To synthesize XG‐stabilized Ag nanoparticles, 10 mL of an aqueous AgNO_3_ solution (3 mM) and 10 mL of an aqueous XG solution (3 mM) were mixed in a conical flask, followed by the addition of 20 mg of PAN/*Cb* nanofibers. The pH of the resulting mixture was adjusted to 7.0, and the solution was stirred at room temperature for 12 hours.

### Morphological, Structural, and Colloidal Characterization

The morphological characteristics of the algae, algal nanofibers, and Ag nanoparticles were examined using scanning electron microscope (SEM) with FEI Quanta FEG 250 and Carl Zeiss 300VP after gold coating. Elemental compositions were analyzed using an energy‐dispersive X‐ray (EDX) detector integrated into the SEM (FEI Quanta FEG 250). The morphology and size of Ag nanoparticles were examined using a transmission electron microscope (Hitachi HT7800 TEM) operating at an accelerating voltage of 100 kV. The average diameters of the nanofibers and nanoparticles were estimated from SEM or TEM micrographs by measuring at least 100 randomly selected nanofibers or particles using the ImageJ software. Algae powders were analyzed using an inverted microscope (Carl Zeiss Axio Vert.A1). Fourier Transform Infrared (FTIR) spectrometer (Perkin Elmer Spectrum 2) was used to investigate the algae, algal nanofibers, and Ag nanoparticles over a wavenumber range of 4000–450 cm⁻¹. The crystallinity of the samples was determined using an X‐ray diffractometer (XRD, Panalytical Empyrean) at a scanning rate of 1°/min over a 5–80° range. UV–vis spectrophotometers, Ocean Optics (DH2000‐BAL, FL, USA) and BMG Labtech (CLARIOstar Plus), were used for absorbance measurements. The zeta potential and particle size of Ag nanoparticles were measured using a Malvern Zetasizer Nano ZS (ZEN 3600) at pH 7.0 and 8.6.

### Antimicrobial Tests

The antimicrobial activity of Ag nanoparticles was assessed against Gram‐negative (*E. coli*) and Gram‐positive (*S. aureus*) bacteria using a modified ASTM E2149 procedure. Bacteria were cultured overnight in nutrient broth (NB) and adjusted to the 0.5 McFarland standard with peptone water. The suspensions were then serially diluted to final concentrations of 2.5 × 10⁴ CFU/mL for *E. coli* and 3.7 × 10⁴ CFU/mL for *S. aureus*. The cultures were incubated at 37 °C for 24 hours, and colony‐forming units (CFU/mL) were counted to determine antimicrobial activity.

### Dye Degradation

The photocatalytic degradation experiments of PAN/*Cb*/Ag@XG nanofibers were performed by comparing MB (5 mL, 5 ppm) under no‐light, day‐ and UV‐light (0.6 mW, 366 nm). The time‐dependent photocatalytic degradation experiments were conducted at pH 7.0, ambient temperature, and with 6 mg of the nanofiber. The reusability of the nanofibers was investigated over three consecutive cycles. After each cycle, the PAN/*Cb*/Ag@XG nanofibers were separated, washed with distilled water, and then reused in the Ag nanoparticle synthesis. The used PAN/*Cb*/Ag@XG nanofibers were re‐exposed to a fresh MB solution in each cycle.

The rate of MB degradation was analyzed using the pseudo‐first‐order kinetic model, as indicated in the Equation.

(1) $$\ln\left(\frac{C_{0}}{C_{t}}\right)=kt$$

where *k* was the rate constant and *C_0_* and *C_t_* were the MB concentration (ppm) at the initial time and at time *t*, respectively.^[^ ^87^ ^]^

## Conflict of Interest

The authors declare no conflict of interest.

## Supporting information



Supporting Information

## Data Availability

The data that support the findings of this study are available from the corresponding author upon reasonable request.
