# Supplementary material for: From Reduction To Remediation: Sustainable Use of Algal Fibrous Mats for Silver Nanoparticle Synthesis and Dye Removal
Source: Macromol Rapid Commun. 2025 Feb 20;46(13):2401033. doi: 10.1002/marc.202401033 (PMC12227230; doi:10.1002/marc.202401033)
Supplement: Supplementary file 1 — Supporting Information [file MARC-46-2401033-s001.docx]

Supporting Information

From Reduction to Remediation: Sustainable Use of Algal Fibrous Mats for Silver Nanoparticle Synthesis and Dye Removal

Fatma Rabia Karaduman, Betül Öztürk Köksal, Ayşegül Ülkü Metin and Nesrin Horzum*


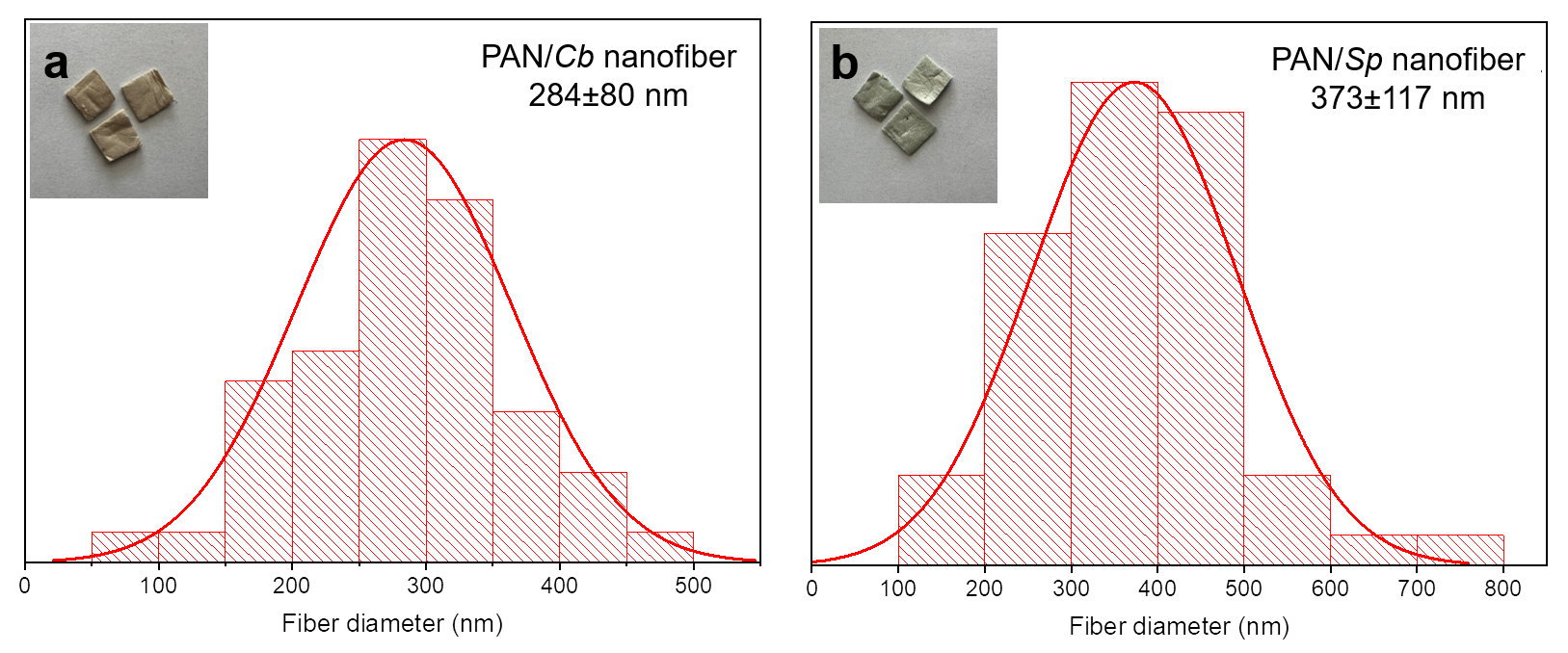


Figure S1. Diameter distributions and their physical appearance of PAN/*Cb* (a) and PAN/*Sp* (b) nanofibers.


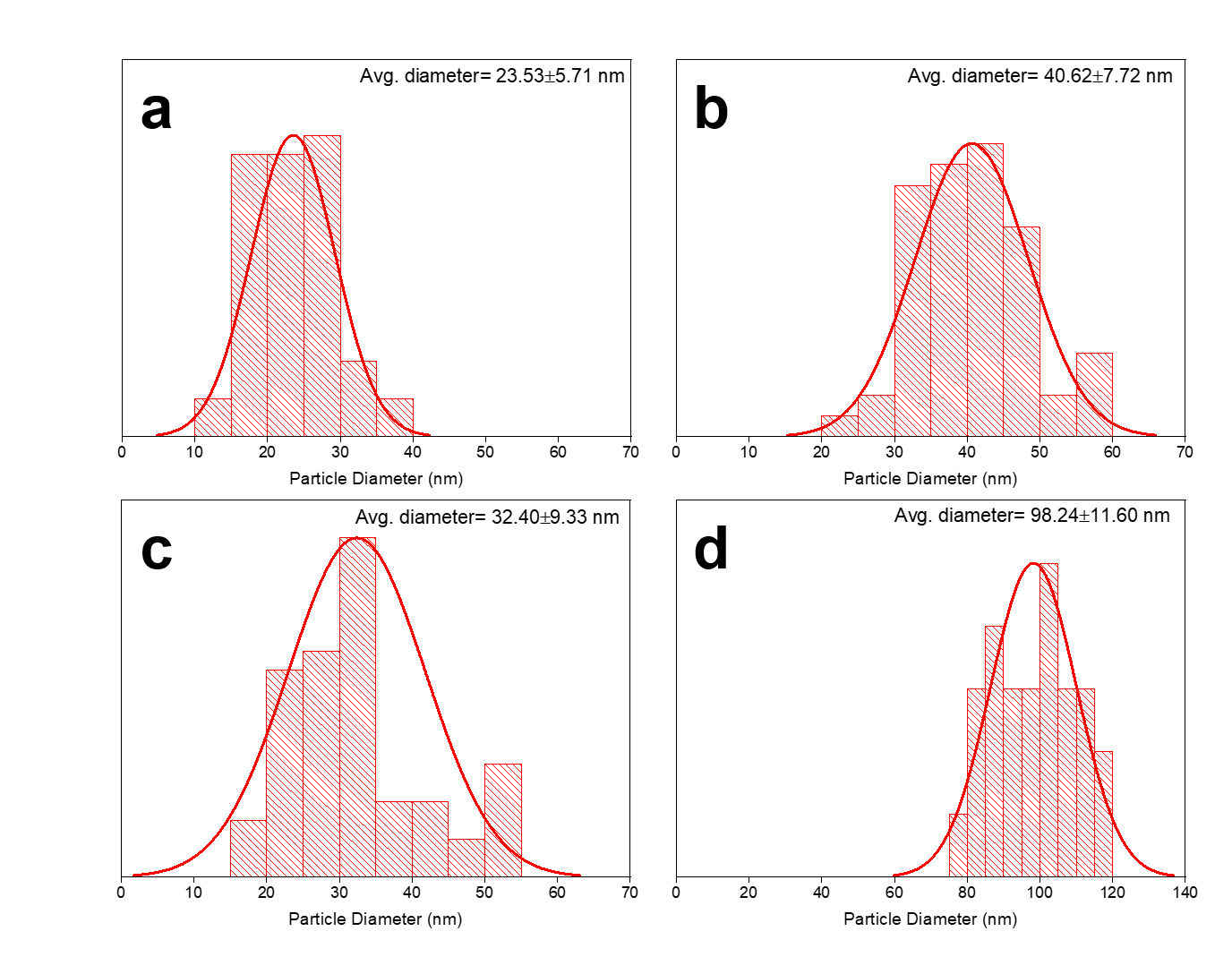


Figure S2. Diameter distributions of Ag nanoparticles obtained from the treatment of powdered *Spirulina* sp. (a) and *C. barbata* (b) (2.0 mg), and nanofibers of PAN/*Sp* (c) and PAN/*Cb* (d) (20 mg) with AgNO₃ solution (20 mL, 3 mM) for 24 hours.

**Figure S3.** The calibration plot of the pseudo-first-order kinetic model for the catalytic degradation of MB under different conditions.
